# Educational Needs for Pressure Injury and Diabetic Foot Care Among Community-Based Nurses in Korea: A Borich and Locus for Focus Analysis

**DOI:** 10.3390/healthcare14203545

**Published:** 2026-10-21

**Authors:** Seungmi Park, Hyeyoung Lee, Je Hyun Kim, Jiye Seo, Eungyeong Choi

**Affiliations:** 1 College of Nursing, Chungbuk National University, Cheongju-si 28644, Chungcheongbuk-do, Republic of Korea; spark2020@chungbuk.ac.kr (S.P.); hyoung28@chungbuk.ac.kr (H.L.); seo0111@korea.kr (J.S.); 2 Cheongju Joo Chan Mi Visiting Nursing Center, Cheongju-si 28696, Chungcheongbuk-do, Republic of Korea; ss6743@naver.com

**Keywords:** pressure ulcer, diabetic foot, needs assessment, continuing nursing education, community health nursing, Republic of Korea

## Abstract

Background/Objectives: Pressure injuries and diabetic foot ulcers are prevalent chronic wounds requiring nurses to provide prevention, assessment, treatment, and education, especially in community settings in Korea. Although knowledge–practice gaps have been reported for both conditions, evidence remains limited on which interventions should be prioritized for education. This study assessed community-based nurses’ perceived importance and self-reported performance of pressure injury management and diabetic foot care interventions to identify educational priorities using the Borich needs assessment and the Locus for Focus model. Methods: A descriptive survey was conducted with 128 registered nurses, fewer than the a priori minimum of 210. Participants rated the perceived importance and self-reported performance of 18 pressure injury interventions and 10 diabetic foot interventions on 3-point scales. Educational priorities were determined using the Borich needs assessment and the Locus for Focus model. Results: Perceived importance was significantly higher than self-reported performance for pressure injury management (1.79 ± 0.29 vs. 1.61 ± 0.47; discrepancy = 0.177; Borich = 0.316; t = 3.968, Cohen’s *d*_z_ = 0.35, *p* < 0.001) and diabetic foot care (1.76 ± 0.31 vs. 1.53 ± 0.51; discrepancy = 0.228; Borich = 0.401; t = 5.143, *d*_z_ = 0.45, *p* < 0.001). First-priority educational needs for pressure injury management centered on treatment interventions and positioning/repositioning, whereas diabetic foot priorities emphasized preventive foot care and patient education. Conclusions: First-priority educational needs were identified separately for pressure injury management and diabetic foot care. The findings can inform the relative emphasis of continuing education content for community-based nurses.

## 1. Introduction

Chronic wounds are major health concerns that require prolonged treatment and ongoing management, reduce patients’ health-related quality of life, and impose a substantial burden on healthcare systems [1]. Pressure injuries can compromise patient health, safety, and quality of life [2], whereas diabetic foot ulcers are serious diabetes-related complications that may lead to infection, lower-extremity amputation, and other adverse outcomes [3]. Early identification of risk factors and appropriate preventive and management strategies are therefore important for reducing adverse health consequences associated with both conditions [2,3].

Nurses play an important role in pressure injury management by assessing risk factors, implementing preventive interventions, and monitoring skin and wound status [2]. In diabetic foot care, nurses assess risk factors and foot status, provide preventive care and patient education, and support ongoing management [4,5]. Chronic wounds often require continued care beyond hospital-based treatment, and wound care services are increasingly provided in community-based settings, including home healthcare and visiting nursing services [6,7]. Community-based nurses therefore need adequate knowledge and clinical competence in wound prevention, assessment, treatment, and patient education to provide safe and continuous chronic wound care.

However, gaps in nurses’ knowledge and practice have been reported in both pressure injury management and diabetic foot care. Studies have identified areas requiring improvement in nurses’ knowledge of pressure injury prevention [2], while gaps in nurses’ knowledge and training related to diabetic foot assessment, preventive care, and patient education have also been reported [4,5]. Nurses’ knowledge of chronic wound management has been shown to vary across clinical settings, suggesting that educational planning should consider both practice context and clinical needs [8]. Educational components within multifaceted pressure injury prevention interventions have also been associated with improvements in nurses’ knowledge and practice [9]. These findings support the need for systematic education in pressure injury management and diabetic foot care.

Effective educational programs should be based not only on whether education is needed but also on which competencies require priority attention. One approach is to examine the discrepancy between perceived importance and self-reported performance. The Borich needs assessment estimates relative educational needs by weighting the discrepancy between perceived importance and performance according to the perceived importance of each item. The Locus for Focus model identifies priority areas by considering both importance and the importance–performance discrepancy. Recent nursing studies have applied these two approaches together to systematically identify educational priorities [10,11], including among home care nurses [11].

Previous studies have demonstrated educational needs in both pressure injury management and diabetic foot care [2,4,5,9]. However, most have examined nurses’ knowledge, attitudes, or practices within each care area separately. Pressure injury management and diabetic foot care were examined within the same study because both represent common chronic wound-related care domains in community-based nursing and require ongoing assessment, preventive care, patient education, and follow-up. Evidence remains limited regarding which nursing interventions should be prioritized for education when both perceived importance and self-reported performance are considered among community-based nurses. Assessing these two dimensions may help identify specific areas in which educational support is most needed. Accordingly, this study aimed to assess community-based nurses’ perceived importance and self-reported performance of pressure injury management and diabetic foot care interventions and to identify educational priorities using the Borich needs assessment and the Locus for Focus model.

## 2. Materials and Methods

### 2.1. Study Design

This descriptive survey was conducted to identify priority educational needs among community-based nurses for nursing interventions related to pressure injury prevention and management and diabetic foot prevention and management.

### 2.2. Participants

Participants were registered nurses working in community-based healthcare settings in Korea. The inclusion criteria were: (1) being a registered nurse; (2) having at least 6 months of total clinical experience; (3) having worked at the current institution for at least 6 months; (4) having worked in the current role for at least 6 months; and (5) understanding the purpose of the study and voluntarily agreeing to participate. Nurses whose primary duties were administrative or managerial rather than direct nursing care were excluded.

Community-based nursing care in Korea is delivered mainly through three systems that differ in legal basis, target populations, and nurse qualification requirements.

First, hospital-based home healthcare is provided under the Medical Service Act to patients discharged from or referred by medical institutions. This service must be delivered by home care advanced practice nurses, who are required to complete a graduate-level advanced practice nursing program and pass a national certification examination [12]. Second, long-term care-based services, provided under the Long-Term Care Insurance Act, are delivered to older adults who have been approved for a long-term care grade, either through visiting nursing or within long-term care facilities; visiting nursing may be provided by nurses with at least 2 years of clinical experience [13]. Third, public health institutions, such as public health centers, provide visiting health management and chronic disease management services mainly to socially and economically vulnerable residents [14].

The sample size was calculated using G*Power version 3.1.9.7 (Heinrich-Heine-Universität Düsseldorf, Düsseldorf, Germany) for a two-tailed paired-samples *t*-test comparing perceived importance and self-reported performance, which was the primary comparison of this study. A minimum sample size of 210 was required, assuming an effect size (*d*_z_) of 0.25, a significance level of 0.05, and statistical power of 0.95. A prior study applying the Borich needs assessment and Locus for Focus model in home visit nursing assumed an effect size of 0.30 for this paired comparison [15]; a more conservative value of 0.25 was adopted in the present study. Considering an anticipated 15% rate of nonresponse or incomplete questionnaires, the target sample size was 248 participants.

### 2.3. Data Collection

Data were collected from 20 June to 27 July 2026 using a structured questionnaire administered either face-to-face or online through a professional survey agency. Online participants were recruited through a recruitment notice posted by the survey agency on an online community bulletin board for community-based nurses. For the face-to-face survey, permission for data collection was obtained from the heads of visiting nursing centers, after which paper questionnaires were distributed to eligible nurses at the centers. Participants completed the questionnaire at a time of their choosing, regardless of working hours, and a small incentive was provided upon completion.

A total of 179 responses were obtained. Two responses with substantial missing data were excluded. Among the online responses, 49 responses showed clearly careless or inattentive response patterns, including selecting the same single response option across the entire questionnaire, and were excluded as invalid. No face-to-face responses were excluded for this reason. The final analytic sample comprised 128 participants: 64 face-to-face respondents and 64 online respondents. As part of the survey agency’s standard data processing procedure, a small number of missing responses in the face-to-face questionnaires were reviewed and imputed before the dataset was provided to the authors; the specific imputation method and the exact number of affected responses were not documented.

Because the total number of nurses who viewed the online recruitment notice or were approached for face-to-face participation was not recorded, a conventional response rate or recruitment reach could not be calculated. Institutional identifiers were not collected to protect participant and institutional anonymity; therefore, the exact number of participating institutions could not be determined.

### 2.4. Measurement

A self-administered questionnaire was used to assess participant characteristics and nursing interventions related to pressure injury prevention and management and diabetic foot prevention and management. For both measures, participants rated each item separately for perceived importance and self-reported performance.

#### 2.4.1. General Characteristics

Participant characteristics included sex, age, educational level, advanced practice nurse (APN) certification, type of institution, employment status, total clinical experience, tenure at the current workplace, the average monthly number of patients receiving chronic wound care, previous chronic wound management education, and the availability of an interprofessional collaboration system at the workplace. APN certification was dichotomized as certified (home care, gerontological, oncology, or hospice APN) versus not certified; five free-text responses describing non-APN roles were classified as not certified. Institution type was consolidated into three categories: home healthcare-based, long-term care-based, and public health institutions. Two respondents who reported both long-term care visiting nursing and a long-term care facility were included in the long-term care-based group. Previous chronic wound management education was recoded as yes versus no from the multiple-response education item.

#### 2.4.2. Pressure Injury Prevention and Management

Nursing interventions related to pressure injury prevention and management were assessed using the practice domain of the Pressure Ulcer Prevention and Treatment Knowledge and Practice instrument developed by Pancorbo-Hidalgo et al. [16] and translated into Korean by Kang [17]. The original instrument consists of knowledge and practice domains; only the practice domain was used in this study because the study focused on educational needs related to nursing practice. The practice domain comprises 18 items: a 12-item prevention component and a 6-item treatment component.

In this study, participants rated each item separately for perceived importance and self-reported performance. Importance was rated on a 3-point scale from 0 (not important) to 2 (very important), and performance was rated on a 3-point scale from 0 (never perform) to 2 (always perform), with higher scores indicating greater perceived importance and more frequent self-reported performance. Kang [17] reported Cronbach’s α values ranging from 0.645 to 0.735 for the practice domain. In this study, Cronbach’s α values for importance and performance were 0.937 and 0.963, respectively, for the full 18-item measure. Mean inter-item correlations were 0.45 for importance and 0.60 for performance; 51% of participants rated all 18 items as very important.

#### 2.4.3. Diabetic Foot Prevention and Management

Nursing interventions related to diabetic foot prevention and management were assessed using 10 practice items used by Kim et al. [18] in a Korean study of care helpers in long-term care facilities. Because the original items were developed for use by care helpers, item wording was minimally modified to reflect the clinical context of nurses. The content validity of the modified items was evaluated by a panel of five experts, comprising four professors of nursing (three specializing in community health nursing and one in adult health nursing) and one visiting nurse. Among them, two were certified wound care specialists, and all held doctoral degrees with a mean of 32 years of clinical or academic experience. Item-level content validity index (I-CVI) values ranged from 0.80 to 1.00, and the scale-level CVI (S-CVI/Ave) was 0.98. Only content validity of the modified instrument was evaluated in this study; structural validity among community-based nurses was not examined.

In this study, participants rated each item separately for perceived importance and self-reported performance. Importance was rated on a 3-point scale from 0 (not important) to 2 (very important), and performance was rated on a 3-point scale from 0 (never perform) to 2 (always perform), with higher scores indicating greater perceived importance and more frequent self-reported performance. Kim et al. [18] used a 1–3 response scale; in this study, the items were administered on a 0–2 scale with the same three response anchors to be consistent with the pressure injury instrument. This difference in scale origin does not affect Cronbach’s α or the paired-samples comparisons of importance and performance (including Cohen’s *d*_z_). However, because the Borich needs score weights the discrepancy by mean importance, it depends on the origin of the importance scale; Borich scores were therefore also calculated on the original 1–3 scale as a sensitivity analysis (Supplementary Table S3). Kim et al. [18] reported a Cronbach’s α of 0.81. In this study, Cronbach’s α was 0.901 for importance and 0.941 for performance. Mean inter-item correlations were 0.48 for importance and 0.62 for performance; 55% of participants rated all 10 items as very important.

### 2.5. Educational Needs Assessment

Educational needs were assessed using the Borich needs assessment and the Locus for Focus model [19,20]. The Borich needs score was calculated for each item as follows:

$$Borich\;needs\;score\;=\frac{\sum \left(I-P\right)\times \bar{I}}{N}$$

where I is the importance rating, P is the performance rating, $\bar{I}$ is the mean importance rating for the item, and N is the number of participants. A higher Borich needs score indicates a greater educational need.

The Locus for Focus model was then used to identify high-priority items. For each scale, mean importance and the mean importance–performance discrepancy were plotted, and their grand means were used as the vertical and horizontal reference lines, respectively. These reference lines formed four quadrants, and items in the high-importance/high-discrepancy (HH) quadrant were considered to have relatively high educational needs. In this classification, “low importance” indicates that an item’s mean importance score is below the overall grand mean and does not imply low clinical importance. Thus, items in the LH quadrant may still represent clinically important aspects of care despite having relatively lower importance scores within the set of items.

Final educational priorities were determined according to the prioritization procedure proposed by Cho [21] and applied by Lee and Jung [15]. Educational priorities for pressure injury management and diabetic foot care were determined separately using the following procedure: (1) the importance–performance discrepancy was examined using paired-samples *t*-tests; (2) Borich needs scores were calculated and ranked; (3) the number of items classified in the HH quadrant of the Locus for Focus model was identified; (4) the same number of items with the highest Borich needs scores was selected; and (5) overlap between the HH-quadrant items and the selected highest-ranked Borich items was examined. Items identified by both methods were classified as first-priority educational needs, whereas items identified by only one method were classified as second-priority educational needs. In this study, an educational priority refers to the relative emphasis that content may warrant in continuing education, based on nurses’ perceived importance and self-reported performance. It does not indicate clinical priority or clinical effectiveness, and whether education on a given intervention improves nursing practice or patient outcomes requires separate evaluation. This procedure was adopted prior to data collection. Setting the number of Borich-selected items equal to the number of HH items allowed the two approaches to select sets of the same size so that their overlap could be examined directly. To examine the sensitivity of this classification to the grand-mean cutoffs, the Locus for Focus classification was repeated using median-based reference lines as a sensitivity analysis (Supplementary Table S4). Because both the Borich needs assessment and the Locus for Focus model are derived from the same underlying importance and performance data, they are not independent methods of validation; agreement between the two approaches reflects convergence between two related summaries of the same data rather than independent confirmation.

### 2.6. Statistical Analysis

Statistical analyses were performed using IBM SPSS Statistics version 21.0 for Windows (IBM Corp., Armonk, NY, USA) and Python version 3.12.13 (Python Software Foundation, Wilmington, DE, USA). Participant characteristics were summarized using frequencies and percentages, and perceived importance and self-reported performance scores were summarized using means and standard deviations. Internal consistency was assessed using Cronbach’s α. Differences in perceived importance and self-reported performance according to participant characteristics were analyzed using independent-samples *t*-tests or one-way analysis of variance, as appropriate, with Scheffé post hoc tests following significant analyses of variance. For two-group comparisons, Welch’s *t*-test was used when the homogeneity-of-variance assumption was violated. Differences between perceived importance and self-reported performance were examined using paired-samples *t*-tests, and Cohen’s *d*_z_ was calculated as the effect size for paired observations. Given the ordinal and ceiling-limited nature of the 3-point response scale, Wilcoxon signed-rank tests were additionally conducted at the item level as a non-parametric check alongside the paired-samples *t*-tests (Supplementary Table S6). To address multiplicity, Holm correction was applied to the 44 subgroup comparisons of participant characteristics with perceived importance and self-reported performance, and to the item-level paired comparisons within each scale. Paired-samples *t*-tests were also used to compare the importance–performance discrepancy between subdomains within each scale. Statistical significance was set at a two-sided *p* < 0.05.

Because the final analytic sample (*n* = 128) was smaller than the a priori minimum, a post hoc power analysis and a sensitivity analysis for the minimum detectable effect size were conducted using G*Power version 3.1.9.7, based on the test for the difference between two dependent means (matched pairs) and a two-tailed significance level of 0.05. Achieved power was 0.802 for the a priori effect size (*d*_z_ = 0.25) and 0.976 and 0.999 for the effect sizes observed for pressure injury management (*d*_z_ = 0.35) and diabetic foot care (*d*_z_ = 0.45), respectively. The sensitivity analysis indicated that the final sample could detect effect sizes of *d*_z_ = 0.250, 0.289, and 0.321 with 80%, 90%, and 95% power, respectively.

### 2.7. Ethical Considerations

The study was conducted in accordance with the Declaration of Helsinki and approved by the Institutional Review Board of Chungbuk National University on 28 April 2026 (IRB No. CBNU-2026-A-0054), prior to data collection. All participants received information on the purpose and procedures of the study, the voluntary nature of participation, the right to withdraw, and the protection of personal information, and informed consent was obtained before participation.

For face-to-face participants, permission for data collection was obtained from the heads of the participating visiting nursing centers before the survey was administered. Online participants were recruited individually rather than through institutional channels, and their participation was based on individual voluntary consent.

## 3. Results

### 3.1. Participant Characteristics

The general characteristics of the study participants are presented in Table 1. Most participants were women (96.1%), and the mean age was 46.68 ± 9.71 years. A bachelor’s degree was the most common educational level (45.3%), and 60.9% did not hold advanced practice nurse (APN) certification. The mean total clinical experience was 13.37 ± 7.07 years, and the mean experience at the current workplace was 5.23 ± 4.36 years. More than half of the participants worked in long-term care-based institutions (52.3%), and 76.6% were employed full-time. In addition, 37.5% had no previous chronic wound management education, and 47.7% reported an interprofessional collaboration system at their workplace. Among the retained participants, the two data collection modes also differed in the institutional composition of respondents: face-to-face respondents were mostly from long-term care-based institutions (49/64), whereas online respondents were mostly from home healthcare-based (36/64) or public health institutions (10/64).

Perceived importance of pressure injury management differed significantly according to APN certification, type of institution, experience at the current workplace, and interprofessional collaboration system, whereas self-reported performance differed according to type of institution, average number of chronic wound patients managed per month, and previous chronic wound management education.

For diabetic foot care, perceived importance differed according to educational level, APN certification, employment status, type of institution, and interprofessional collaboration system, whereas self-reported performance differed according to type of institution, average number of chronic wound patients managed per month, and previous chronic wound management education. After applying Holm correction for the 44 comparisons, eight associations remained significant (Table 2): APN certification with perceived importance in both domains; type of institution with perceived importance in pressure injury management and with self-reported performance in both domains; the average number of chronic wound patients managed per month with self-reported performance in both domains; and interprofessional collaboration with perceived importance of pressure injury management. The associations with educational level, employment status, current workplace experience, and previous chronic wound management education did not remain significant after correction (Supplementary Table S5). Post hoc analyses showed that self-reported performance was significantly lower in public health institutions than in home healthcare-based and long-term care-based institutions for both pressure injury management and diabetic foot care. Nurses managing no chronic wound patients also reported significantly lower performance than all other patient-volume groups. No significant differences in perceived importance or self-reported performance were observed according to sex, age, or total clinical experience.

### 3.2. Importance, Performance, and Educational Needs by Scale and Item Group

For pressure injury management, perceived importance was significantly higher than self-reported performance (1.79 ± 0.29 vs. 1.61 ± 0.47; *t* = 3.968, *p* < 0.001, Cohen’s *d*_z_ = 0.35). The importance–performance discrepancy was 0.177, and the Borich needs score was 0.316. Treatment interventions showed a significantly larger discrepancy (0.253 vs. 0.139; t = 4.86, *p* < 0.001) and Borich needs score (0.453 vs. 0.248) than preventive interventions. Perceived importance was significantly higher than self-reported performance for both preventive nursing interventions (*t* = 3.120, *p* = 0.002, Cohen’s *d*_z_ = 0.28) and treatment nursing interventions (*t* = 5.198, *p* < 0.001, Cohen’s *d*_z_ = 0.46).

For diabetic foot care, perceived importance was significantly higher than self-reported performance (1.76 ± 0.31 vs. 1.53 ± 0.51; *t* = 5.143, *p* < 0.001, Cohen’s *d*_z_ = 0.45). The importance–performance discrepancy was 0.228, and the Borich needs score was 0.401. Prevention and education showed a larger discrepancy (0.237 vs. 0.215) and a higher Borich needs score (0.419 vs. 0.376) than assessment, documentation, and reporting, but the difference was not statistically significant (t = 0.83, *p* = 0.41)**.** Perceived importance was significantly higher than self-reported performance for both prevention and education (*t* = 5.121, *p* < 0.001, Cohen’s *d*_z_ = 0.45) and assessment, documentation, and reporting (*t* = 4.649, *p* < 0.001, Cohen’s *d*_z_ = 0.41) (Table 3).

### 3.3. Priority Educational Needs

**Figure 1:**
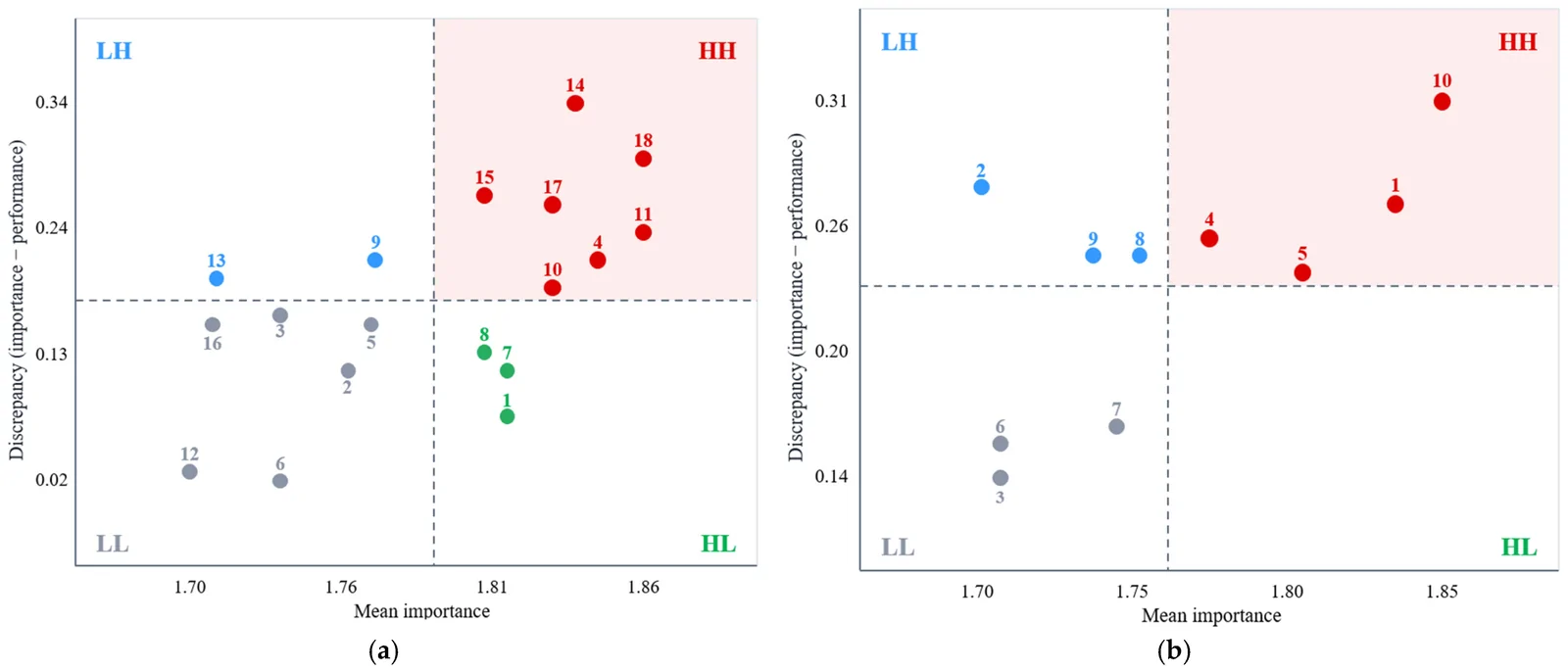
Locus for Focus model of educational needs (*N* = 128): (**a**) pressure injury management and (**b**) diabetic foot care. Numbers correspond to items in Supplementary Tables S1 and S2. HH, high importance/high discrepancy; HL, high importance/low discrepancy; LH, low importance/high discrepancy; LL, low importance/low discrepancy. The light pink-shaded area indicates the high importance/high discrepancy (HH) quadrant, representing first-priority educational needs.

For pressure injury management, seven items were classified in the HH quadrant of the Locus for Focus model (items 4, 10, 11, 14, 15, 17, and 18) (Figure 1a). Six of these items were also among the seven highest-ranked Borich items and were classified as first-priority educational needs: hydrogel-assisted autolytic debridement, dead-space filling or packing, antiseptic use, moisture-retentive dressing application, 30° positioning, and 2 h repositioning. Because current guidance recommends individualized repositioning rather than a fixed interval [22], the 2 h repositioning item should not be interpreted as indicating a performance deficit; its priority ranking may instead reflect a need for education on repositioning assessment and decision-making. Transfer equipment use and nutritional assessment were identified by only one of the two methods and were classified as second-priority educational needs (Table 4).

For diabetic foot care, four items were classified in the HH quadrant (items 1, 4, 5, and 10) (Figure 1b). Three of these items were also among the four highest-ranked Borich items and were classified as first-priority educational needs: foot hygiene assessment and education, use of socks and shoes outdoors, and appropriate water temperature for foot washing. Checking shoes for foreign objects and assessing foot skin moisture were identified by only one of the two methods and were classified as second-priority educational needs (Table 4). When the Borich scores were recalculated using the original 1–3 scale, the five highest-ranked items and the first-priority classification remained unchanged; a minor exchange of ranks 6 and 7 occurred between items 5 and 9, neither of which was classified as first priority under either scaling (Supplementary Table S3). Complete item-level results are provided in Supplementary Tables S1 and S2.

### 3.4. Sensitivity Analyses

To examine the robustness of the overall discrepancies and the priority classification, several sensitivity analyses were conducted (Supplementary Table S4). Twenty-three participants (18.0%) gave the same rating across every pressure injury and diabetic foot item (22 of whom rated every item at the maximum score and 1 at the middle score); because such uniform responses were plausible given the overall high importance ratings observed in this sample, they were retained in the primary analysis rather than excluded, and their influence was examined as part of the sensitivity analyses below. Among nurses who managed at least one chronic wound patient per month (n = 107), the overall pressure injury discrepancy was small and not statistically significant (0.048; *d*_z_ = 0.17; *p* = 0.080), whereas the diabetic foot discrepancy remained significant (0.124; *d*_z_ = 0.33; *p* = 0.001); item-level priority classifications were unchanged under this restriction. Restricting the analysis to nurses outside public health institutions produced similar results for pressure injury management; for diabetic foot care, items 4 and 5 exchanged first- and second-priority classifications. Excluding the 23 participants with uniform responses across all items increased the overall discrepancies (pressure injury 0.215; diabetic foot 0.278) but did not change the priority classification. Using median-based reference lines for the Locus for Focus model instead of grand means left five of six first-priority pressure injury items and all three first-priority diabetic foot items unchanged; one pressure injury item (antiseptic use) moved to second priority, and the second-priority items differed slightly. Excluding the fixed 2 h repositioning item from the pressure injury classification did not change the remaining first- and second-priority items.

## 4. Discussion

### 4.1. Principal Findings

This study identified educational needs for pressure injury management and diabetic foot care among community-based nurses using the Borich needs assessment and the Locus for Focus model. Perceived importance was significantly higher than self-reported performance in both care domains. The overall discrepancies corresponded to small-to-moderate within-participant differences (*d*_z_ = 0.35 and 0.45 for pressure injury management and diabetic foot care, respectively). For individual priority items, 24–34% of nurses rated importance higher than performance, indicating that these discrepancies, while modest on average, were concentrated among a substantial minority of nurses rather than reflecting a uniform pattern across the sample. For pressure injury management, treatment interventions showed a larger importance–performance discrepancy and a higher Borich needs score than preventive interventions. For diabetic foot care, prevention and education showed a numerically larger discrepancy and Borich needs score than assessment, documentation, and reporting, although this difference was not statistically significant. First-priority educational needs for pressure injury management focused on debridement, dead-space management, dressing application, positioning and repositioning, and wound infection assessment and antimicrobial or antiseptic selection, whereas diabetic foot priorities focused on preventive foot care and patient education. These findings indicate the educational focus identified within each care domain.

### 4.2. Pressure Injury Education: Strengthening Treatment Interventions and Clinical Judgment

Previous studies have reported gaps in nurses’ pressure injury knowledge [23] and improvements in pressure injury knowledge and management following structured education [24]. In this study, the highest-priority educational areas included debridement, dead-space management, dressing application, positioning and repositioning, and the appropriate assessment of wound infection and evidence-based selection of antimicrobial or antiseptic approaches. These findings highlight treatment-related areas that may warrant greater emphasis in continuing education for community-based nurses.

The importance–performance discrepancies identified in this study should be interpreted as indicators of potential educational priorities rather than direct evidence of deficient clinical competence or inadequate prior education. A low level of self-reported performance may reflect several factors beyond educational exposure, including limited opportunities to encounter specific clinical conditions, differences in patient case mix, scope of practice, institutional protocols, staffing, and access to appropriate wound-care resources. Such discrepancies may also reflect recall or social desirability bias. Consistent with this interpretation, among nurses who managed at least one chronic wound patient per month, the overall pressure injury discrepancy was small and not statistically significant, although item-level priorities were unchanged (Section 3.4; Supplementary Table S4). This consideration is particularly relevant in community-based settings, where nurses may have substantially different responsibilities and opportunities to perform pressure injury or diabetic foot care.

Accordingly, the high-priority items identified through the Borich and Locus for Focus analyses should be viewed as areas that may warrant greater emphasis in continuing education, rather than as evidence that nurses lack the knowledge or competence required to perform these practices. Further studies using objective competency assessments and measures of clinical exposure are needed to determine whether the identified educational priorities correspond to actual competency gaps and to clarify the factors contributing to lower performance.

The identified priorities should not be interpreted as recommending routine use of these interventions for all patients. Current international guidance recommends individualized repositioning based on mobility and activity, skin and tissue tolerance, clinical condition, comfort, treatment goals, and the pressure-redistribution properties of the support surface [22]. Accordingly, the fixed 2 h repositioning item should be interpreted as a need for education on repositioning assessment and decision-making rather than as a universal schedule. Similar caution applies to other intervention-specific items. Debridement, dead-space packing, and antiseptic or antimicrobial use depend on wound assessment, physician orders, institutional protocols, and the nurse’s scope of practice. Their high priority therefore indicates a need for education on assessment and decision-making about when these interventions are appropriate, rather than a recommendation for their routine or universal use. Second-priority needs included the use of transfer equipment to avoid dragging and assessment of overall nutritional status. Because implementation in community settings may also be affected by workload, equipment availability, communication, and team support [25], pressure injury education should integrate wound assessment, treatment principles, pressure redistribution, nutritional risk, and clinical judgment in selecting and applying interventions.

### 4.3. Diabetic Foot Education: Strengthening Prevention and Education

The first-priority educational needs for diabetic foot care were checking foot hygiene practices and providing education, teaching and checking the use of socks and shoes when going outdoors, and teaching the use of appropriately warm water for foot washing. These findings are consistent with previous research reporting gaps in nurses’ education regarding footwear and socks [26] and with current diabetic foot guidelines emphasizing regular foot care education, appropriate footwear, and risk-based foot assessment [27,28,29]. The highest Borich needs score was observed for checking foot hygiene practices and providing education. Previous studies have linked factors related to foot care with self-care behaviors among people with a history of diabetic foot ulcers [30], and structured foot care and nurse-led self-management education have shown beneficial effects on patients’ knowledge, self-efficacy, and self-care behaviors [31,32,33]. These findings support education that enables community-based nurses to assess patients’ foot care practices and provide education based on identified needs. However, because these studies reported patient-level outcomes, they do not provide direct evidence that nurse education improves patient behavior.

The second-priority educational needs showed different patterns across the two priority analyses. Checking the inside of shoes for foreign objects before use was classified in the LH quadrant despite a relatively large importance–performance discrepancy, whereas assessing foot skin moisture and intervening when needed was classified in the HH quadrant but ranked sixth in the Borich needs assessment. Importantly, classification in the LH quadrant reflects an importance score below the grand mean within this set of items and should not be interpreted as indicating low clinical importance; checking shoes for foreign objects remains an important preventive component of diabetic foot care. These findings illustrate that the Borich needs assessment and the Locus for Focus model emphasize different aspects of the same importance and performance data when educational priorities are identified. These findings show that the Borich needs assessment and the Locus for Focus model provide complementary information for identifying educational priorities. Because importance ratings were high with relatively little variation, the Borich ranking closely followed the discrepancy ranking; consequently, the agreement between the Borich and Locus for Focus approaches does not represent independent confirmation, as both are derived from the same importance and performance data.

### 4.4. Implications for Educational Program Development and Implementation

Nurses with previous chronic wound management education reported higher self-reported performance in univariable comparisons; however, this association did not remain significant after correction for multiple testing (Supplementary Table S5), and performance also differed according to type of institution and average number of chronic wound patients managed per month. These findings suggest that institutional context and clinical exposure may be more closely associated with performance than prior education alone; because this study was cross-sectional, causal relationships cannot be inferred.

Educational programs should reflect the different priorities identified for each care domain. Pressure injury education should emphasize treatment principles and clinical judgment for selecting and applying interventions, whereas diabetic foot education should emphasize preventive foot care and patient education. The first- and second-priority educational needs should be used to guide the relative emphasis of educational content rather than as a fixed sequence for curriculum delivery. Case-based or practical learning may be useful when opportunities to perform specific interventions are limited. Whether case-based, simulation-based, or digital formats are effective for addressing these priorities among community-based nurses should be examined in future research [34,35,36,37]. Workload, equipment and resources, communication, and referral pathways should also be considered when implementing educational programs in community-based settings [25,38,39].

### 4.5. Limitations

This study has several limitations. First, convenience recruitment and the final analytic sample of 128 participants, which was smaller than the a priori minimum of 210, limit generalizability and may reduce statistical power and precision, particularly for subgroup analyses. However, a post hoc power analysis indicated adequate power (≥0.80) for the a priori and observed effect sizes in the primary paired comparisons, and a sensitivity analysis indicated that the final sample could detect effect sizes as small as *d*_z_ = 0.25–0.32 across 80–95% power; subgroup analyses with small cell sizes (e.g., public health institutions, n = 10) nonetheless have limited power and precision. Second, self-reported performance may be influenced by recall, social desirability, clinical exposure, and local practice context and does not directly measure observed clinical competence. Third, participation lower than targeted may have reflected community-based nurses’ high workload and frequent off-site visits, as well as the small, geographically dispersed nature of the participating institutions. All 49 excluded online responses showed clearly careless responding, with participants selecting the same single response option across the entire questionnaire; no face-to-face responses were excluded for this reason. Face-to-face and online respondents also differed in institutional composition, which may partly reflect lower participation among nurses in long-term care-based institutions for the online survey, or other mode-related differences in who chose to participate. Fourth, the 3-point response scale may have restricted variability and contributed to ceiling effects. Fifth, a small number of missing responses in the face-to-face surveys were imputed by the research agency before the dataset was provided, but the affected cells were not identified, preventing sensitivity analysis of the imputation. Sixth, the diabetic foot items were derived from a measure originally used with care helpers in long-term care facilities; although item wording was modified for the nursing context and content validity was evaluated by an expert panel, further psychometric evaluation of construct validity in community-based nurses is warranted. Several diabetic foot items also combined two actions within a single statement (e.g., checking foot hygiene practices and providing education), so a single rating cannot indicate which component drove the discrepancy or the educational need. Pilot testing or cognitive interviewing with nurses was not conducted when the items were modified. Some pressure injury items also reflect the wording of the source instrument, including a fixed 2 h repositioning interval, whereas current guidance emphasizes individualized repositioning [22]. The high Cronbach’s α values, together with mean inter-item correlations exceeding the range generally considered optimal for performance ratings and the ceiling effects noted above, may reflect that most of the assessed interventions are considered essential nursing practice, as well as possible item redundancy or uniform responding; the present data cannot distinguish between these explanations. Seventh, institutional identifiers were not collected to protect participant anonymity, so the number of participating institutions could not be determined and clustering of nurses within institutions could not be modeled; standard errors may therefore be underestimated to the extent that responses were correlated within institutions. Finally, pressure injury management and diabetic foot care were assessed using different instruments; direct comparison of absolute Borich needs scores between the two scales should therefore be avoided. The findings should be interpreted as priorities within the specific nursing interventions assessed in each domain.

## 5. Conclusions

This study identified domain-specific educational priorities for pressure injury management and diabetic foot care among community-based nurses using the Borich needs assessment and the Locus for Focus model. Pressure injury priorities centered on treatment-related interventions and positioning/repositioning, whereas diabetic foot priorities focused on routine preventive foot care and patient education. The identified priorities indicate where continuing education content may be relatively emphasized, based on perceived importance and self-reported performance; they do not represent clinical priorities or evidence that education on these items will improve patient outcomes, which would require separate evaluation. For community-based care institutions, continuing education on these priorities could be accompanied by institutional support, such as access to wound-care resources, clear wound-care protocols, and consultation or referral pathways, particularly in settings where nurses have limited exposure to chronic wound care. Future surveys could include a “not applicable” response option to distinguish limited opportunity from limited competence. Future research should also include psychometric validation of the modified diabetic foot instrument among community-based nurses, such as factor analysis and cognitive interviewing.

## Figures and Tables

**Table 1:** General characteristics of study participants (*N* = 128).

Variable	Category	n (%) or M ± SD
Sex	Male	5 (3.9)
	Female	123 (96.1)
Age (years)	<40	36 (28.1)
	40–49	45 (35.2)
	≥50	47 (36.7)
	Mean ± SD	46.68 ± 9.71
Education level	Associate degree	29 (22.7)
	Bachelor’s degree	58 (45.3)
	≥Master’s degree	41 (32.0)
Advanced practice nurse certification	Certified	50 (39.1)
	Not certified	78 (60.9)
Type of institution	Home healthcare-based	51 (39.8)
	Long-term care-based	67 (52.3)
	Public health institution	10 (7.8)
Employment status	Full-time	98 (76.6)
	Part-time	28 (21.9)
	Other	2 (1.6)
Total clinical experience (years)	<10	35 (27.3)
	10–19	67 (52.3)
	≥20	26 (20.3)
	Mean ± SD	13.37 ± 7.07
Experience at current workplace (years)	<3	45 (35.2)
	3–5	42 (32.8)
	≥6	41 (32.0)
	Mean ± SD	5.23 ± 4.36
Average number of chronic wound patients managed per month	None	21 (16.4)
	1–2	25 (19.5)
	3–5	44 (34.4)
	6–10	15 (11.7)
	≥11	23 (18.0)
Previous chronic wound management education	Yes	80 (62.5)
	No	48 (37.5)
Interprofessional collaboration system in workplace	Yes	61 (47.7)
	No	45 (35.2)
	Not sure	22 (17.2)

Values are presented as *n* (%) or mean ± SD. Exact *p*-values, effect sizes, and multiplicity-adjusted results for the associations between these characteristics and perceived importance and self-reported performance are provided in Supplementary Table S5.

**Table 2:** Participant characteristics significantly associated with perceived importance or self-reported performance after Holm correction for multiple testing (*N* = 128).

Variable	Pressure Injury				Diabetic Foot			
	Importance		Performance		Importance		Performance	
	t/F	d/η^2^ (*p*)	t/F	d/η^2^ (*p*)	t/F	d/η^2^ (*p*)	t/F	d/η^2^ (*p*)
APN certification	−4.77	0.86 (<0.001)	−0.26 †	0.04 (1.000)	−3.59	0.65 (0.017)	−0.76 †	0.12 (1.000)
Type of institution	9.50	0.13 (0.006)	81.22	0.57 (<0.001)	4.46	0.07 (0.473)	32.72	0.34 (<0.001)
Chronic wound patient volume	0.78	0.02 (1.000)	14.02	0.31 (<0.001)	0.39	0.01 (1.000)	9.16	0.23 (<0.001)
Interprofessional collaboration	8.95	0.13 (0.009)	0.39	0.01 (1.000)	4.07	0.06 (0.599)	0.78	0.01 (1.000)

APN = advanced practice nurse. For the two-group comparison (APN certification), the statistic (*t*/*F* column) is an independent-samples *t*-test and the effect size is Cohen’s *d*; for comparisons with three or more groups, the statistic is a one-way analysis-of-variance *F* and the effect size is eta squared (η^2^). † Welch’s *t*-test (equal variances not assumed). *p*-values are Holm-adjusted across all 44 comparisons of participant characteristics; values are capped at 1.000. Complete results for all 44 comparisons, including variables not shown in this table, are provided in Supplementary Table S5.

**Table 3:** Importance, performance, and educational needs for pressure injury management and diabetic foot care (*N* = 128).

Scale	Item Group	Items	Importance, M ± SD	Performance, M ± SD	Discrepancy	t	*p*	*d*_z_		Borich Needs Score
								M	95% CI	
Pressure injury	Preventive interventions	12	1.79 ± 0.31	1.65 ± 0.46	0.139	3.120	0.002	0.28	0.13, 0.41	0.248
	Treatment interventions	6	1.79 ± 0.29	1.54 ± 0.54	0.253	5.198	<0.001	0.46	0.34, 0.57	0.453
	Total	18	1.79 ± 0.29	1.61 ± 0.47	0.177	3.968	<0.001	0.35	0.22, 0.48	0.316
Diabetic foot	Prevention and education	6	1.77 ± 0.32	1.53 ± 0.53	0.237	5.121	<0.001	0.45	0.33, 0.58	0.419
	Assessment, documentation, and reporting	4	1.75 ± 0.34	1.53 ± 0.52	0.215	4.649	<0.001	0.41	0.27, 0.55	0.376
	Total	10	1.76 ± 0.31	1.53 ± 0.51	0.228	5.143	<0.001	0.45	0.33, 0.58	0.401

Discrepancy = importance − performance; M = mean; *d*_z_ = Cohen’s effect size for paired samples; 95% CI = 95% confidence interval.

**Table 4:** Priority educational needs identified using the Borich needs assessment and the Locus for Focus model (*N* = 128).

Scale	Priority	Item	Borich	Rank	LfF
Pressure injury	First	14. Use hydrogel to promote autolytic debridement of necrotic tissue	0.631	1	HH
	First	18. Lightly fill or pack dead space when present	0.552	2	HH
	First	15. Use an antiseptic solution when signs of infection are present	0.479	3	HH
	First	17. Apply a moisture-retentive dressing (e.g., hydrocolloid, alginate, hydrogel, foam, or hydrofiber)	0.471	4	HH
	First	11. When elevating the upper body, elevate the lower limbs by 30° and use a 30° lateral tilt	0.436	5	HH
	First	4. Reposition bedbound patients at 2 h intervals	0.389	6	HH
	Second	9. Use transfer equipment and lift rather than drag the patient	0.372	7	LH
	Second	10. Assess overall nutritional status in patients at risk of pressure injury	0.343	8	HH
Diabetic foot	First	10. Check foot-hygiene practices and provide education	0.579	1	HH
	First	1. Teach and check the use of socks and shoes when going outdoors	0.488	2	HH
	First	4. Teach the use of appropriately warm water for foot washing	0.443	4	HH
	Second	2. Check the inside of shoes for foreign objects before use	0.464	3	LH
	Second	5. Assess foot-skin moisture and intervene when needed	0.423	6	HH

LfF = Locus for Focus; HH = high importance/high discrepancy; LH = low importance/high discrepancy. First = identified by both methods; Second = identified by one method.

## Data Availability

The data presented in this study are available on request from the corresponding author due to privacy restrictions related to participant survey responses.

## Supplementary Materials

The following supporting information can be downloaded at: https://www.mdpi.com/article/10.3390/healthcare14203545/s1, Table S1: Item-level educational needs assessment for pressure injury management; Table S2: Item-level educational needs assessment for diabetic foot care; Table S3: Borich needs scores for diabetic foot care items calculated on the administered 0–2 scale and the original 1–3 scale; Table S4: Sensitivity analyses of the overall discrepancy and priority classification; Table S5: Importance and performance scores by participant characteristic, with test statistics, effect sizes, and Holm-adjusted *p*-values for all 44 comparisons; Table S6: Item-level paired comparisons with Holm adjustment and Wilcoxon signed-rank tests; STROBE checklist; CHERRIES checklist [40].

## Abbreviations

The following abbreviations are used in this manuscript:

HH	High importance and high discrepancy
HL	High importance and low discrepancy
LfF	Locus for Focus
LH	Low importance and high discrepancy
LL	Low importance and low discrepancy
SD	Standard deviation
APN	Advanced practice nurse
