# Supplementary material for: Educational Needs for Pressure Injury and Diabetic Foot Care Among Community-Based Nurses in Korea: A Borich and Locus for Focus Analysis
Source: Healthcare (Basel). 2026 Oct 21;14(20):3545. doi: 10.3390/healthcare14203545 (PMC13704309; doi:10.3390/healthcare14203545)
Supplement: Supplementary file 1 [file healthcare-14-03545-s001.zip › CHERRIES_Checklist_1005.pdf]

CHERRIES Checklist (Checklist for Reporting Results of Internet E-Surveys) — Online Survey Component

Manuscript: "Educational Needs for Pressure Injury and Diabetic Foot Care among Community-Based Nurses in Korea: A Borich and Locus for Focus Analysis"

Item	Checklist Item	Reported on (Section) / Response
**Design**		
1	Describe survey design	Cross-sectional descriptive survey administered face-to-face (paper) or online (web-based), described in Section 2.1 and 2.3. This checklist addresses the online component only.
**Development and Pre-testing**		
2	Developed the instrument de novo, or modified an existing instrument, and whether it was tested	The pressure injury and diabetic foot instruments were adapted from existing Korean-validated tools (Section 2.4.2–2.4.3). Pilot testing or cognitive interviewing of the online questionnaire was not conducted (Section 4.5).
**Recruitment Process and Description of the Sample Having Access to the Questionnaire**		
3	Open survey (accessible to anyone) vs. closed survey (accessible only to a sample the investigators controlled)	Open survey: accessible to any community-based nurse who viewed the recruitment notice (Section 2.3)
4	How did participants access the e-survey (e.g., direct URL, link from another site, pop-up)? Was the survey advertised?	Participants accessed the survey via a link included in a recruitment notice posted on an online community bulletin board for community-based nurses (Section 2.3)
5	How were potential participants identified and how were they approached? Was a sampling frame used?	No defined sampling frame was used; recruitment relied on nurses viewing the publicly posted notice (Section 2.3). The number of nurses who viewed the notice was not recorded, so reach and response rate could not be calculated (Section 2.3; Section 4.5)
**Survey Administration**		
6	Web/E-mail	Web-based questionnaire (online survey platform administered by the professional survey agency)
7	Context: Was the survey e-mail or the website visited stand-alone, or associated with a web portal? What website/forum?	Survey link posted on a community bulletin board for community-based nurses; not embedded in a larger portal
8	Mandatory/voluntary: Was participation voluntary or coerced (e.g., as part of employment)? Was an incentive offered?	Participation was voluntary; a small incentive was provided upon completion (Section 2.3)
9	Incentives: Was any incentive offered (e.g., prize, money, gift)?	A small incentive was provided upon completion (Section 2.3)

Item	Checklist Item	Reported on (Section) / Response
10	Time/date: In what time period were data collected?	20 June to 27 July 2026 (Section 2.3)
11	Randomization of items or questionnaires: To prevent response bias, items or item blocks or alternative questionnaires can be presented in random order	Items were presented in a fixed order; randomization was not used
12	Adaptive questioning: To reduce number and complexity of questions, adaptive questioning may be used	Not used; all eligible respondents received the same fixed set of items
13	Number of items: Indicate the number of questionnaire items per page	18 pressure injury items and 10 diabetic foot items, each rated for importance and performance (56 ratings total), presented across the online questionnaire (Section 2.4.2–2.4.3)
14	Number of screens (pages): Indicate number of web pages used for the entire questionnaire	Not recorded by the authors; administered and hosted by the survey agency
15	Completeness check: Was there a technical facility (e.g., JavaScript) for automatic completeness checks?	Not documented; the survey agency’s standard data-processing procedure included a manual missing-data verification step (Section 2.3)
16	Review step: Were respondents able to review and change their answers (e.g., with a Back button, edit function, or summary page) before submitting?	Not documented by the survey agency
**Response Rates**		
17	Usability/pre-testing: Were usability and technical functionality tested before fielding?	Not documented
18	Response rate: Define how the response rate was calculated. Participation (view rate), completion rate (ratio of users who finished the survey)	A conventional response rate could not be calculated because the number of nurses who viewed the online recruitment notice was not recorded (Section 2.3; Section 4.5). Of 113 online responses received, 49 were excluded for careless responding, yielding 64 analyzed online responses (Section 2.3)
**Preventing Multiple Entries from the Same Individual**		
19	Attempts to mitigate multiple entries from the same individual (e.g., cookies, IP address check, registration)	No technical measures (e.g., cookies, IP-address checks, unique identifiers) were used; instead, responses showing clearly careless or inattentive patterns, including selecting the same single response option across the entire questionnaire, were identified and excluded by the survey agency (Section 2.3). This is acknowledged as a limitation (Section 4.5)
**Analysis**		
20	Handling of incomplete questionnaires: Were only completed questionnaires analyzed, or also partial responses?	Responses with substantial missing data were excluded (2

Item	Checklist Item	Reported on (Section) / Response
		responses); a small number of remaining missing values in the face-to-face data were imputed by the survey agency (Section 2.3)
21	Questionnaires submitted with an atypical timestamp: Were submission timestamps analyzed to identify duplicate or low-quality responses?	Not analyzed by the authors; the survey agency identified careless responses based on response patterns rather than timestamps (Section 2.3)
22	Statistical correction: Statistical correction for multiple testing, non-normal distributions, etc.	Holm correction was applied to subgroup and item-level comparisons, and non-parametric Wilcoxon signed-rank tests were used alongside paired t-tests (Section 2.6)

Note: This checklist applies to the online data-collection component of the mixed-mode survey (online and face-to-face).
