# Supplementary material for: Educational Needs for Pressure Injury and Diabetic Foot Care Among Community-Based Nurses in Korea: A Borich and Locus for Focus Analysis
Source: Healthcare (Basel). 2026 Oct 21;14(20):3545. doi: 10.3390/healthcare14203545 (PMC13704309; doi:10.3390/healthcare14203545)
Supplement: Supplementary file 1 [file healthcare-14-03545-s001.zip › STROBE_Checklist_1005.pdf]

STROBE Statement—Checklist of items that should be included in reports of cross-sectional studies

Manuscript: "Educational Needs for Pressure Injury and Diabetic Foot Care among Community-Based Nurses in Korea: A Borich and Locus for Focus Analysis"

	Item No.	Recommendation	Reported on (Section)
**Title and abstract**			
	1(a)	Indicate the study's design with a commonly used term in the title or the abstract	Title ("...A Borich Needs Assessment and Locus for Focus Analysis"); Abstract (Methods: "A descriptive survey...")
	1(b)	Provide in the abstract an informative and balanced summary of what was done and what was found	Abstract
**Introduction**			
	2	Explain the scientific background and rationale for the investigation being reported	Section 1 (Introduction)
	3	State specific objectives, including any prespecified hypotheses	Section 1, final paragraph
**Methods**			
	4	Present key elements of study design early in the paper	Section 2.1 (Study Design)
	5	Describe the setting, locations, and relevant dates, including periods of recruitment, exposure, follow-up, and data collection	Sections 2.2–2.3 (Participants; Data Collection)
	6(a)	Give the eligibility criteria, and the sources and methods of selection of participants	Section 2.2 (Participants); Section 2.3 (recruitment channels)
	6(b)	(Not applicable — no matching was used)	Not applicable
	7	Clearly define all outcomes, exposures, predictors, potential confounders, and effect modifiers. Give diagnostic criteria, if applicable	Sections 2.4.1–2.4.3 (Measurement)
	8*	For each variable of interest, give sources of data and details of methods of assessment (measurement). Describe comparability of assessment methods if there is more than one group	Sections 2.4.1–2.4.3
	9	Describe any efforts to address potential sources of bias	Section 2.3 (exclusion of careless responses); Section 4.5 (Limitations)
	10	Explain how the study size was arrived at	Section 2.2 (sample size calculation)
	11	Explain how quantitative variables were handled in the analyses. If applicable, describe which groupings were chosen and why	Section 2.6 (Statistical Analysis)
	12(a)	Describe all statistical methods, including those used to control for confounding	Section 2.6
	12(b)	Describe any methods used to examine subgroups and interactions	Section 2.6 (subgroup comparisons); Section 3.4 (Sensitivity Analyses)
	12(c)	Explain how missing data were addressed	Section 2.3 (imputation by survey agency); Section 4.5 (Limitations)
	12(d)	If applicable, describe analytical methods taking account of sampling	Not applicable (convenience sample;

	Item No.	Recommendation	Reported on (Section)
		strategy	see Section 4.5)
	12(e)	Describe any sensitivity analyses	Section 3.4 (Sensitivity Analyses); Supplementary Table S4
**Results**			
	13(a)	Report the numbers of individuals at each stage of the study — e.g., numbers potentially eligible, examined for eligibility, confirmed eligible, included in the study, completing follow-up, and analysed	Section 2.3 (179 responses → 128 analytic sample)
	13(b)	Give reasons for non-participation at each stage	Section 2.3 (missing data, careless responding); Section 4.5
	13(c)	Consider use of a flow diagram	Not included — recruitment and exclusion flow is described in Section 2.3
	14(a)	Give characteristics of study participants (e.g., demographic, clinical, social) and information on exposures and potential confounders	Table 1; Section 3.1
	14(b)	Indicate the number of participants with missing data for each variable of interest	Section 2.3 (dataset provided to authors contained no missing values; imputation performed by survey agency, see Section 4.5)
	14(c)	(Not applicable — no follow-up)	Not applicable
	15*	Report numbers of outcome events or summary measures	Table 2; Table 3
	16(a)	Give unadjusted estimates and, if applicable, confounder-adjusted estimates and their precision (e.g., 95% confidence interval). Make clear which confounders were adjusted for and why they were included	Table 2 (95% CI for dz); Table S5 (subgroup comparisons)
	16(b)	Report category boundaries when continuous variables were categorized	Table 1
	16(c)	If relevant, consider translating estimates of relative risk into absolute risk for a meaningful time period	Not applicable
	17	Report other analyses done — e.g., analyses of subgroups and interactions, and sensitivity analyses	Section 3.4; Table 2; Supplementary Tables S3–S6
**Discussion**			
	18	Summarise key results with reference to study objectives	Section 4.1
	19	Discuss limitations of the study, taking into account sources of potential bias or imprecision. Discuss both direction and magnitude of any potential bias	Section 4.5
	20	Give a cautious overall interpretation of results considering objectives, limitations, multiplicity of analyses, results from similar studies, and other relevant evidence	Sections 4.1–4.4
	21	Discuss the generalisability (external validity) of the study results	Section 4.5
**Other information**			

	Item No.	Recommendation	Reported on (Section)
	22	Give the source of funding and the role of the funders for the present study and, if applicable, for the original study on which the present article is based	Funding statement (back matter)
