# Supplementary material for: Educational Needs for Pressure Injury and Diabetic Foot Care Among Community-Based Nurses in Korea: A Borich and Locus for Focus Analysis
Source: Healthcare (Basel). 2026 Oct 21;14(20):3545. doi: 10.3390/healthcare14203545 (PMC13704309; doi:10.3390/healthcare14203545)
Supplement: Supplementary file 1 [file healthcare-14-03545-s001.zip › Supplementary_Materials_S1-S6.pdf]

Supplementary Materials

Educational Needs for Pressure Injury and Diabetic Foot Care among Community-Based Nurses in Korea: A Borich and Locus for Focus Analysis

Table S1. Item-level educational needs assessment for pressure injury management (N = 128).

Item	Educational content	Importance, M ± SD	Performance, M ± SD	Discrepancy	t	p	dz	Borich	Rank	LfF
1	Assess pressure-injury risk using a validated tool at admission or first contact	1.81 ± 0.39	1.73 ± 0.52	0.078	1.45	0.150	0.13	0.142	16	HL
2	Assess skin at pressure-prone sites (e.g., sacrum and heels)	1.76 ± 0.43	1.64 ± 0.57	0.117	1.94	0.054	0.17	0.206	15	LL
3	Apply barrier cream, moisturizer, or protective film to pressure-prone sites	1.73 ± 0.46	1.57 ± 0.61	0.164	2.51	0.013	0.22	0.285	10	LL
4	Reposition bedbound patients at 2-h intervals	1.84 ± 0.41	1.63 ± 0.60	0.211	3.33	0.001	0.29	0.389	6	HH
5	Change position hourly when seated in a chair or wheelchair	1.77 ± 0.43	1.61 ± 0.59	0.156	2.44	0.016	0.22	0.276	11	LL
6	Use a pressure-redistributing support surface (e.g., air mattress)	1.73 ± 0.44	1.71 ± 0.55	0.023	0.39	0.698	0.03	0.041	18	LL
7	Use pillows or cushions to relieve pressure	1.81 ± 0.41	1.70 ± 0.57	0.117	1.88	0.063	0.17	0.212	14	HL
8	Offload the heels using heel elevation and a pillow under the calves	1.80 ± 0.45	1.67 ± 0.59	0.133	2.17	0.032	0.19	0.240	13	HL
9	Use transfer equipment and lift rather than drag the patient	1.77 ± 0.43	1.55 ± 0.61	0.211	3.33	0.001	0.29	0.372	7	LH
10	Assess overall nutritional status in patients at risk of pressure injury	1.83 ± 0.38	1.64 ± 0.59	0.188	3.40	<0.001	0.30	0.343	8	HH
11	When elevating the upper body, elevate the lower limbs by 30° and use a 30° lateral tilt	1.86 ± 0.37	1.62 ± 0.60	0.234	4.03	<0.001	0.36	0.436	5	HH
12	Educate the primary caregiver (family or paid caregiver) about pressure-injury prevention	1.70 ± 0.48	1.67 ± 0.56	0.031	0.54	0.588	0.05	0.053	17	LL
13	Have an appropriately trained professional remove necrotic tissue using sterile instruments	1.71 ± 0.46	1.52 ± 0.63	0.195	3.27	0.001	0.29	0.334	9	LH
14	Use hydrogel to promote autolytic debridement of necrotic tissue	1.84 ± 0.37	1.49 ± 0.63	0.344	5.71	<0.001	0.50	0.631	1	HH
15	Use an antiseptic solution when signs of infection are present	1.80 ± 0.40	1.54 ± 0.65	0.266	4.14	<0.001	0.37	0.479	3	HH
16	Use a topical antimicrobial when signs of infection are present	1.71 ± 0.47	1.55 ± 0.63	0.156	2.33	0.021	0.21	0.267	12	LL
17	Apply a moisture-retentive dressing (e.g., hydrocolloid, alginate, hydrogel, foam, or hydrofiber)	1.83 ± 0.38	1.57 ± 0.61	0.258	4.72	<0.001	0.42	0.471	4	HH
18	Lightly fill or pack dead space when present	1.86 ± 0.35	1.56 ± 0.64	0.297	5.21	<0.001	0.46	0.552	2	HH

Values are mean ± SD. Discrepancy = importance − performance. Borich = mean discrepancy × mean importance. dz = Cohen's effect size for paired samples. LfF = Locus for Focus; HH = high importance/high discrepancy; HL = high importance/low discrepancy; LH = low importance/high discrepancy; LL = low importance/low discrepancy. Multiplicity-adjusted and non-parametric p-values are reported in Table S6.

Table S2. Item-level educational needs assessment for diabetic foot care (N = 128).

Item	Educational content	Importance, M ± SD	Performance, M ± SD	Discrepancy	t	p	dz	Borich	Rank	LfF
1	Teach and check the use of socks and shoes when going outdoors	1.84 ± 0.37	1.57 ± 0.64	0.266	4.65	<0.001	0.41	0.488	2	HH
2	Check the inside of shoes for foreign objects before use	1.70 ± 0.46	1.42 ± 0.67	0.273	4.18	<0.001	0.37	0.464	3	LH
3	Assess and report corns or wounds on the feet	1.70 ± 0.49	1.56 ± 0.64	0.141	2.22	0.028	0.20	0.240	10	LL
4	Teach the use of appropriately warm water for foot washing	1.77 ± 0.44	1.52 ± 0.61	0.250	4.34	<0.001	0.38	0.443	4	HH
5	Assess foot-skin moisture and intervene when needed	1.80 ± 0.40	1.57 ± 0.58	0.234	4.27	<0.001	0.38	0.423	6	HH
6	Teach and check the selection of appropriate socks and footwear	1.70 ± 0.46	1.55 ± 0.61	0.156	2.60	0.011	0.23	0.266	9	LL
7	Teach drying between the toes after foot washing	1.74 ± 0.44	1.58 ± 0.61	0.164	2.81	0.006	0.25	0.286	8	LL
8	Assess foot temperature and perfusion; document and report abnormalities	1.75 ± 0.43	1.51 ± 0.65	0.242	3.88	<0.001	0.34	0.424	5	LH
9	Systematically assess and document foot status	1.73 ± 0.46	1.49 ± 0.64	0.242	3.82	<0.001	0.34	0.420	7	LH
10	Check foot-hygiene practices and provide education	1.85 ± 0.36	1.54 ± 0.65	0.312	5.25	<0.001	0.46	0.579	1	HH

Values are mean ± SD. Discrepancy = importance − performance. Borich = mean discrepancy × mean importance. dz = Cohen's effect size for paired samples. LfF = Locus for Focus; HH = high importance/high discrepancy; LH = low importance/high discrepancy; LL = low importance/low discrepancy. Multiplicity-adjusted and non-parametric p-values are reported in Table S6.

Table S3. Borich needs scores for diabetic foot care items calculated on the administered 0–2 scale and the original 1–3 scale (N = 128).

Item	Mean importance (0–2)	Mean discrepancy	Borich (0–2)	Rank	Borich (1–3)	Rank	LfF
10	1.852	0.312	0.579	1	0.891	1	HH
1	1.836	0.266	0.488	2	0.754	2	HH
2	1.695	0.273	0.464	3	0.737	3	LH
4	1.773	0.250	0.443	4	0.693	4	HH
8	1.750	0.242	0.424	5	0.666	5	LH
5	1.805	0.234	0.423	6	0.657	6	HH
9	1.734	0.242	0.420	7	0.662	7	LH
7	1.742	0.164	0.286	8	0.450	8	LL
6	1.703	0.156	0.266	9	0.422	9	LL
3	1.703	0.141	0.240	10	0.381	10	LL

Borich (1–3) = mean discrepancy × (mean importance + 1). Locus for Focus (LfF) quadrants are identical on both scales because all items and the reference line shift equally.

Table S4. Sensitivity analyses of the overall discrepancy and priority classification.

Analysis	n	PI discrepancy (dz, p)	PI first	PI second	DF discrepancy (dz, p)	DF first	DF second
All participants	128	0.177 (0.35, <0.001)	4, 11, 14, 15, 17, 18	9, 10	0.228 (0.45, <0.001)	1, 4, 10	2, 5
≥1 chronic wound patient/month	107	0.048 (0.17, 0.080)	4, 11, 14, 15, 17, 18	9, 10	0.124 (0.33, 0.001)	1, 4, 10	2, 5
Excluding public health institutions	118	0.070 (0.22, 0.017)	4, 11, 14, 15, 17, 18	9, 10	0.140 (0.36, <0.001)	1, 5, 10	2, 4
Both restrictions	106	0.047 (0.17, 0.087)	4, 11, 14, 15, 17, 18	9, 10	0.124 (0.32, 0.001)	1, 5, 10	2, 4
Excluding uniform responders	105	0.215 (0.39, <0.001)	4, 11, 14, 15, 17, 18	9, 10	0.278 (0.51, <0.001)	1, 4, 10	2, 5
Median reference lines	128	—	4, 11, 14, 17, 18	10, 15	—	1, 4, 10	2, 8
Excluding item 4 (2-h repositioning)	128	—	11, 14, 15, 17, 18	9, 10	—	—	—

PI = pressure injury; DF = diabetic foot. Discrepancy values are M (dz, p). Uniform responders gave the same rating to all items (n = 23). Item numbers correspond to Tables S1 and S2. — indicates a value not computed for that analysis.

Table S5. Importance and performance scores by participant characteristic, with test statistics, effect sizes, and Holm-adjusted p-values for all 44 comparisons (N = 128).

Variable	Category	n	PI importance		PI performance		DF importance		DF performance	
			M ± SD	t/F; d/η^2 (p)	M ± SD	t/F; d/η^2 (p)	M ± SD	t/F; d/η^2 (p)	M ± SD	t/F; d/η^2 (p)
Sex	Male	5	1.81 ± 0.29	−0.19<br>0.08 (1.000)	1.78 ± 0.19	−0.81<br>0.37 (1.000)	1.74 ± 0.40	0.14<br>0.06 (1.000)	1.40 ± 0.46	0.59<br>0.27 (1.000)
	Female	123	1.79 ± 0.29		1.60 ± 0.48		1.76 ± 0.31		1.54 ± 0.51	
Age (years)	<40	36	1.79 ± 0.31	2.78<br>0.04 (1.000)	1.57 ± 0.55	0.26<br>0.00 (1.000)	1.74 ± 0.34	1.98<br>0.03 (1.000)	1.53 ± 0.57	0.01<br>0.00 (1.000)
	40–49	45	1.71 ± 0.30		1.65 ± 0.35		1.70 ± 0.32		1.52 ± 0.47	
	≥50	47	1.85 ± 0.26		1.60 ± 0.51		1.83 ± 0.28		1.54 ± 0.51	
Education level	Associate degree	29	1.83 ± 0.32	3.03<br>0.05 (1.000)	1.46 ± 0.53	1.98<br>0.03 (1.000)	1.79 ± 0.35	4.21<br>0.06 (0.546)	1.36 ± 0.57	2.19<br>0.03 (1.000)
	Bachelor's degree	58	1.83 ± 0.28		1.65 ± 0.53		1.82 ± 0.27		1.60 ± 0.55	
	≥Master’s degree	41	1.70 ± 0.27		1.66 ± 0.28		1.65 ± 0.32		1.56 ± 0.38	
Advanced practice nurse certification	Certified	50	1.65 ± 0.25	−4.77<br>0.86 (<0.001)	1.62 ± 0.23	−0.26†<br>0.04 (1.000)	1.64 ± 0.26	−3.59<br>0.65 (0.017)	1.57 ± 0.27	−0.76†<br>0.12 (1.000)
	Not certified	78	1.88 ± 0.28		1.60 ± 0.57		1.84 ± 0.32		1.51 ± 0.62	
Type of institution	Home healthcare-based	51	1.66 ± 0.30	9.50<br>0.13 (0.006)	1.63 ± 0.28	81.22<br>0.57 (<0.001)	1.66 ± 0.32	4.46<br>0.07 (0.473)	1.56 ± 0.32	32.72<br>0.34 (<0.001)
	Long-term care-based	67	1.87 ± 0.24		1.77 ± 0.29		1.83 ± 0.29		1.66 ± 0.46	
	Public health institution	10	1.86 ± 0.33		0.43 ± 0.54		1.79 ± 0.38		0.52 ± 0.55	
Employment status	Full-time	98	1.76 ± 0.29	2.04<br>0.03 (1.000)	1.58 ± 0.51	1.03<br>0.02 (1.000)	1.72 ± 0.32	3.13<br>0.05 (1.000)	1.53 ± 0.54	0.59<br>0.01 (1.000)
	Part-time	28	1.87 ± 0.28		1.68 ± 0.31		1.87 ± 0.29		1.50 ± 0.42	
	Other	2	2.00 ± 0.00		1.94 ± 0.08		2.00 ± 0.00		1.90 ± 0.14	
Total clinical experience (years)	<10	35	1.81 ± 0.31	0.27<br>0.00 (1.000)	1.54 ± 0.61	0.61<br>0.01 (1.000)	1.77 ± 0.34	0.74<br>0.01 (1.000)	1.44 ± 0.63	0.92<br>0.01 (1.000)
	10–19	67	1.79 ± 0.29		1.64 ± 0.44		1.78 ± 0.31		1.58 ± 0.45	
	≥20	26	1.75 ± 0.27		1.63 ± 0.31		1.69 ± 0.28		1.52 ± 0.47	
Experience at current workplace (years)	<3	45	1.86 ± 0.26	4.33<br>0.06 (0.500)	1.66 ± 0.42	1.57<br>0.02 (1.000)	1.78 ± 0.35	0.71<br>0.01 (1.000)	1.52 ± 0.51	1.63<br>0.03 (1.000)
	3–5	42	1.69 ± 0.33		1.66 ± 0.45		1.71 ± 0.30		1.64 ± 0.44	
	≥6	41	1.81 ± 0.25		1.50 ± 0.53		1.79 ± 0.29		1.44 ± 0.56	
Chronic wound patients per month	None	21	1.87 ± 0.30	0.78<br>0.02 (1.000)	1.04 ± 0.79	14.02<br>0.31 (<0.001)	1.75 ± 0.39	0.39<br>0.01 (1.000)	0.99 ± 0.74	9.16<br>0.23 (<0.001)
	1–2	25	1.81 ± 0.33		1.82 ± 0.23		1.82 ± 0.31		1.68 ± 0.39	
	3–5	44	1.77 ± 0.25		1.73 ± 0.22		1.76 ± 0.27		1.66 ± 0.31	
	6–10	15	1.78 ± 0.25		1.59 ± 0.32		1.70 ± 0.31		1.54 ± 0.36	
	≥11	23	1.73 ± 0.35		1.68 ± 0.28		1.74 ± 0.36		1.61 ± 0.46	
Previous chronic wound management education	Yes	80	1.76 ± 0.28	1.32<br>0.24 (1.000)	1.71 ± 0.26	−2.61†<br>0.57 (0.417)	1.75 ± 0.29	0.43<br>0.08 (1.000)	1.63 ± 0.32	−2.54†<br>0.55 (0.473)
	No	48	1.83 ± 0.30		1.45 ± 0.66		1.78 ± 0.35		1.36 ± 0.69	
Interprofessional collaboration system	Yes	61	1.68 ± 0.28	8.95<br>0.13 (0.009)	1.60 ± 0.37	0.39<br>0.01 (1.000)	1.68 ± 0.28	4.07<br>0.06 (0.599)	1.59 ± 0.34	0.78<br>0.01 (1.000)
	No	45	1.90 ± 0.25		1.65 ± 0.53		1.86 ± 0.30		1.50 ± 0.64	
	Not sure	22	1.86 ± 0.30		1.55 ± 0.59		1.77 ± 0.38		1.44 ± 0.61	

PI = pressure injury; DF = diabetic foot; APN = advanced practice nurse. For two-group comparisons (sex, APN certification, previous chronic wound education), the statistic is an independent-samples t-test and the effect size is Cohen’s d; for comparisons with three or more groups, the statistic is a one-way analysis-of-variance F and the effect size is eta squared (η^2). † Welch’s t-test (equal variances not assumed). p-values are Holm-adjusted across all 44 comparisons; values are capped at 1.000.

Table S6. Item-level paired comparisons with Holm adjustment and Wilcoxon signed-rank tests (N = 128).

(a) Pressure injury management

Item	p (paired t-test)	Holm-adjusted p	Wilcoxon p
1	0.150	0.449	0.141
2	0.054	0.272	0.053
3	0.013	0.121	0.012
4	0.001	0.014	0.001
5	0.016	0.130	0.015
6	0.698	1.000	0.654
7	0.063	0.272	0.058
8	0.032	0.190	0.033
9	0.001	0.014	0.001
10	<0.001	0.012	0.001
11	<0.001	0.001	<0.001
12	0.588	1.000	0.543
13	0.001	0.014	0.001
14	<0.001	<0.001	<0.001
15	<0.001	<0.001	<0.001
16	0.021	0.148	0.018
17	<0.001	<0.001	<0.001
18	<0.001	<0.001	<0.001

(b) Diabetic foot care

Item	p (paired t-test)	Holm-adjusted p	Wilcoxon p
1	<0.001	<0.001	<0.001
2	<0.001	<0.001	<0.001
3	0.028	0.028	0.027
4	<0.001	<0.001	<0.001
5	<0.001	<0.001	<0.001
6	0.011	0.021	0.010
7	0.006	0.017	0.006
8	<0.001	<0.001	<0.001
9	<0.001	<0.001	<0.001
10	<0.001	<0.001	<0.001

Item numbers and wording, t statistics, and effect sizes are reported in Tables S1 and S2. Holm correction was applied within each scale (18 pressure injury items; 10 diabetic foot items).
